# Impact of COVID pandemic upon radiological services in a tertiary care hospital - A clinical audit

**DOI:** 10.12669/pjms.38.6.5272

**Published:** 2022

**Authors:** Laiba Masood, Salma Gul, Suraya Bano, Rohama Saeed

**Affiliations:** 1 Dr. Laiba Masood, MBBS. Resident, Radiology Department, Shifa International Hospital, Islamabad, Pakistan; 2 Dr. Salma Gul, FRCR, MBBS. Consultant, Radiology Department, Shifa International Hospital, Islamabad, Pakistan; 3 Dr. Suraya Bano, FCPS, MBBS. Consultant, Radiology Department, Radiology Department, Shifa International Hospital, Islamabad, Pakistan; 4 Dr. Rohama Saeed, MBBS. Resident, Radiology Department. Radiology Department, Shifa International Hospital, Islamabad, Pakistan

**Keywords:** Radiology, Workflow, COVID-19

## Abstract

**Background and Objectives:**

Radiology emerged as one of the frontline clinical services in the COVID-19 pandemic. This audit study aimed to evaluate the impact of COVID-19 on the radiological services in a tertiary care hospital in terms of workload and case mix.

**Methods:**

We compared the overall workload of the radiology department between March 2019 to December 2020, emphasizing the number of CTs and Chest radiographs performed during the pandemic. The first period starting when the first confirmed case presented to our hospital and the second control period in the same months in 2019. The imaging parameters included the total number of CTs, MRIs, Ultrasounds, Radiographs, CTs from the emergency room (ER), OPD, IPD, CT chest performed for COVID-19 and other emergency indications. All parameters were calculated by taking average each month in both study periods.

**Results:**

An overall decrease was observed in the number of all primary imaging modalities during the pandemic, with ultrasound showing a maximum reduction in numbers (36.5%) followed by radiographs (29.6%) and MRIs (13.8%) compared to 2019. However, total CTs showed a minimal decrease of 1.6% with a significant leap in HRCTs performed reaching up to 80.5%.

**Conclusion:**

COVID-19 and resultant movement restrictions, although they did lead to a reduction in overall radiology work volume, were compensated by an increase in the number of studies performed through emergency and for management of COVID-19 infection.

**Abbreviations:**
COVID-19: = Coronavirus disease 2019
CT: = Computed Tomography
HRCT: = High resolution CT
MRI: = Magnetic Resonance Imaging
USG: = Ultrasound
CXR: = Chest Radiograph
RIS: = Radiology Information system
ER: = Emergency Department
IPD: = In patient Department
OPD: = Outpatient Department

## INTRODUCTION

COVID-19 pandemic presented the healthcare system with an enormous, unprecedented challenge in diagnosis, treatment, and management.^1^-^3^ Radiological departments worldwide rapidly became one of the pillars in triaging patients affected by this global pandemic with the help of imaging. HRCTs were the primary diagnostic modality, CXRs for screening and follow up of COVID-19 and ultrasound provided the bedside imaging.^2^,^4^,^5^ While out-patient departments and elective procedures were purposely closed worldwide, there was a significant decrease in the workload of all medical specialties.^1^,^6^ However, there was still a large load of patients presenting through ER to diagnose and manage COVID-19.^2^ Even though each region’s response varied from other, social and economic lockdowns were observed globally as an effort to nip this virus transmission in the bud. Despite the availability of vaccines, new surges are still being observed with the prospect of more infected patients presenting to hospitals.^3^ Whilst routine clinical work had been suspended initially, COVID-19 pandemic still took a huge toll on the medical community especially in our country due to limited resources, labor shortage despite virus infection themselves.^2^,^5^ The purpose of this audit study was to understand the impact of COVID-19 on the overall workload of the radiology department in a tertiary care setup and compare the type of examinations performed during and after the government-imposed lockdown during the COVID-19 pandemic as opposed to the previous year.

## METHODS

This retrospective audit study was conducted at Radiology Department of Shifa International Hospital, Islamabad, Pakistan from March 2019 to December 2020. Approval of hospital Institutional Review Board and Ethics Committee (Ref: IRB# 092-21/ Dated; April 9, 2021) was obtained. We compared and calculated the overall workload in our radiology department regarding concerned imaging modalities, emphasizing CT scans and chest radiography performed during the COVID-19 pandemic. We selected two study periods for this audit. The first period started from March 2020, when the first COVID-19 was reported in our hospital, till December 2020. The second control period was selected in the same months one year before the first case reported i-e in 2019. We pulled out records from the RIS using specific codes allotted for each examination. The parameters for our audit study included the following primary imaging modalities CT, MRI, ultrasound, and radiographs. We also included the total number of CTs performed from ER, OPD (out-patient) and IPD (in-patient), HRCT performed for COVID-19, and other emergency indications (pulmonary/ vascular angiography, trauma, and abdominal emergencies, etc.). In OPD CTs, patients with oncological workups and follow-ups were also included. Fluoroscopic and mammographic studies make up a lesser volume of our routine workload; therefore, we excluded these studies.

First, we obtained the total number of studies performed for each of the modalities as mentioned above. Then, all imaging parameters were compared and calculated by taking an average of each month during each study period. Finally, we used percentage analysis for the interpretation of primary data using Microsoft excel 365. Furthermore, we paid special attention to lockdown months that is April to July 2020 and compared them to previous year. It was during these months of initial total lockdown that OPDs were closed and emergency department was extended to cater a larger number. of patients. The staff in our radiology department including doctors, technicians and nursing aides, was also divided into two teams with a weekly rota following the rest of departments of hospital. However, afterwards in subsequent COVID waves and government imposed smart lockdowns, the working of our department was continued on as normal routine and we catered all patients who were in need of our services whether routine or emergency under strict SOPs.

## RESULTS

**Fig.1:**
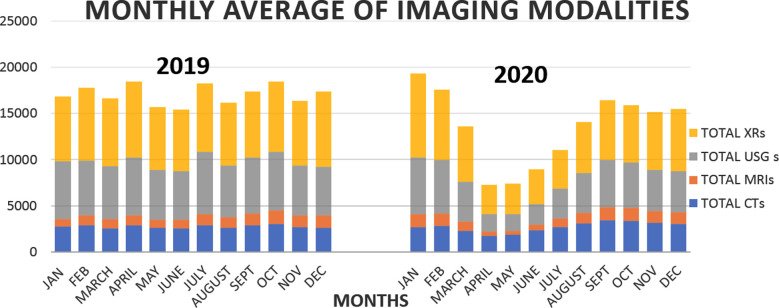
Comparison of monthly total numbers primary imaging modalities during COVID (2020) and pre- COVID (2019).

**Fig.2:**
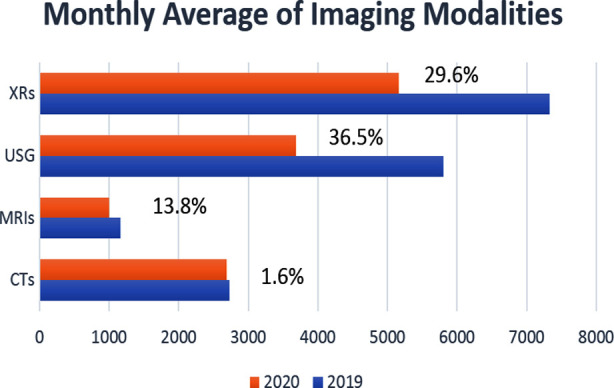
Comparison of monthly average % decrease in primary imaging modalities during COVID (2020) and pre- COVID (2019).

We observed a decrease in the number of all primary imaging modalities during the COVID-19 pandemic, worst in the first wave from 1^st^ April – 30^th^ May 2020 in the government-imposed lockdown. Overall, the audit showed a decrease of 36.5% in the number of ultrasounds, 29.6% in radiographs (XRs), followed by MRIs having a 13.8% reduction compared to 2019 (Fig.1 and Fig.2).

**Fig.3:**
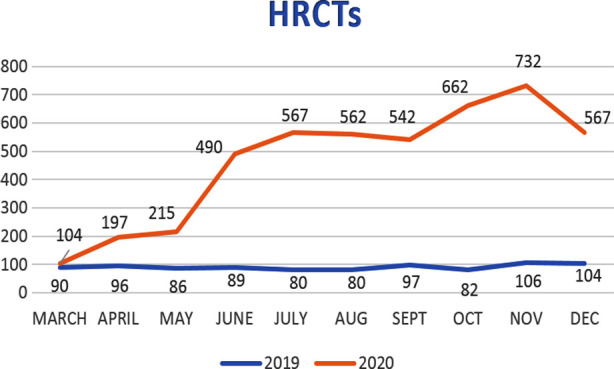
Total no. of HRCTs (high resolution CT) performed in each month for both study periods.

However, only a 1.6% decrease was observed in the total number of CTs with a significant leap in HRCTs performed (80.3%) (Fig.2). We also observed a significant increase in the number of CTs performed through ER and IPDs as due to government imposed OPD closure, patients were only entertained for emergency purposes (Table-I). A large volume of these CTs were HRCTs performed for screening, diagnosis, and management of COVID-19 (Fig.3).

**Table I:** Monthly average breakdown of CTs and Radiographs with percentage increase/ decrease during COVID and Pre-COVID era.

Modality	Pre-COVID Period (2019)	COVID Period (2020)	% increase/ decrease
IPD CTs	1223	1402.2	12.7% increase
OPD CTs	1499	1266.2	15.5% decrease
ER CTs	489.1	799.6	38.7% increase
HRCTs	91	463.8	80.5% increase
Trauma related CTs	65.2	53.6	17.8% decrease
CT pulmonary angiography	34.1	38.5	11.4% increase
CT chest, abdomen, and pelvis	130.1	121.7	6.4% increase
IPD Radiographs	4252.2	3375.2	20.6% decrease
OPD Radiographs	3076.8	1781.8	62.6% decrease
Chest Radiographs	4804.7	3862.2	19.6% decrease

However, we saw a moderate influx of CTs performed through OPDs after the Pakistani government lifted the movement restriction order. These also included oncological patients who had not been able to visit for their regular follow-ups. However, oncology patients with emergent indications were evaluated through ER even when there was lockdown. We also observed a reduction in the trauma-related indications during the COVID-19 era (17.8%) (Table-I). Patients who required CT pulmonary angiography were slightly increased in the COVID-19 study period, 11.4% increase, probably related to hypercoagulability caused by the virus.^3^

**Fig.4:**
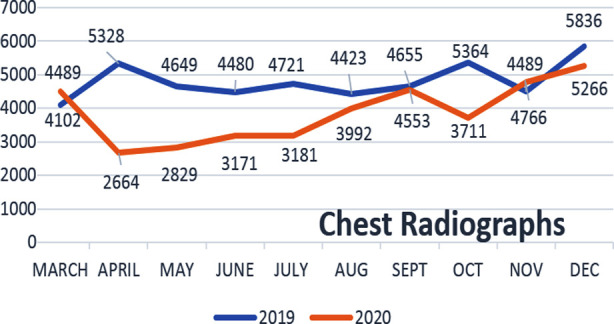
Total no. of chest radiographs performed in each month for both study periods.

Radiography showed a consistent downward trend in IPD and OPD referrals (Table-I). Chest radiographs also showed an initial decline as a significant volume in our department comes for pre-employment and preoperative medical assessments along with routine OPD indications. Once CXRs were included as a part of the COVID-19 screening protocol, this was followed by an increase in CXR numbers during the COVID-19 pandemic (Fig.4). This was introduced in the month of August 2020 after the temporary closure of one of our CT scanners for maintenance. However, the use of CXRs solely for screening of COVID had its own limitations and we have used CT again as primary screening tool for COVID. Primarily, this was due to similar appearance of COVID pneumonia as few other diseases like pulmonary edema, pneumonias caused by other viruses/bacteria, pulmonary hemorrhage etc. on CXR that we could not rely on its findings. So, thereafter it was only used in a selected number of patients who were young or known to have no previous comorbids. This effect can be seen in (Fig.3) with a temporary rise in CXR numbers during Aug-Sept 2020 followed by subsequent decline. The numbers again showed an upward trend during Nov 2020 onwards, however that is more related to lifting of government-imposed lockdown and international travel bans.

## DISCUSSION

Radiology had a pivotal role and formed one of the main pillars in the foundation of COVID-19 pandemic management.^1^,^2^ Amongst other medical specialties, radiological departments had a substantial burden worldwide despite the closure of out-patient departments and postponement of elective surgical procedures.^3^,^4^ In the initial months of the COVID-19 pandemic in Pakistan, starting from March 2020, movement restrictions were imposed to encourage social distancing and reduce the rapid spreading of this deadly virus. Our hospital rapidly developed a workflow system with infrastructure modifications and closure of OPDs, and extension of ER services. We divided our radiological workforce into two teams comprising radiologists, residents, technical staff, and nursing aides with alternate shifts. This resulted in a decrease in overall monthly working hours, however as the infection rate increased, the work burden upon individual teams also multiplied since the usual work volume was now being managed by half of the team.

Overall, during March to August 2020 there was a notable declining trend in the number of all the imaging investigations in comparison to numbers recorded in previous year of 2019 in same months, with the least inkling shift seen in number of CTs. We observed a significant increase in CTs performed through our ER department relative to other imaging modalities, for screening, diagnosis, and management of COVID-19 apart from other routine emergencies, including strokes, abdominal emergencies, trauma, and vascular angiographies. Oncological patients were also given priority in these referrals. An overall increase in the number of CTs performed through emergency does not necessarily mean an increase in positive cases, reflecting that the radiological services guaranteed critical and life-saving imaging despite being faced with this unpredicted crisis.^6^,^7^ Surveys performed worldwide showed a significant downward trend in the workload of primary imaging modalities during the initial lockdown months, which was comparable to our audit results.^3^-^5^

A significant reduction was also noted in the burden of radiography during the initial phase of the pandemic since a large volume of CXRs at our hospital is performed for medical fitness, executive, and preoperative assessments along with routine OPD. This was partly due to the closure of visa processing and international flights along with no new employment opportunities.^8^ However once there was a moderate increase in CXR numbers after the introduction of CXRs as a part of the screening and triaging of COVID-19 patients. In addition, ultrasound which forms a large volume of our OPD services, underwent a significant decline, i-e 36.5%, the largest percentage of all imaging modalities. In contrast, a slight falling trend was noted in the MRI workload.

Following the lifting of lockdown and resumption of our out-patient services, we observed a sudden increase in the volume of imaging studies, even though these numbers do not entirely match those seen in the previous year, probably due to people still feeling fearful of visiting hospitals and avoiding catching the virus.^5^,^7^ These included several oncological referrals, new patients, elective and cosmetic surgeries, and those on follow-ups as well as other indications generally seen in our department.

We did observe a significant improvement in the appropriateness for the use of imaging during COVID pandemic as the referring physicians carefully weighed the necessity of diagnostic imaging in each patient compared to pre-pandemic era. However, we do not have reliable data to confirm that imaging done during lockdown months had more positive findings compared to previous year as this was a retrospective study.

In our literature review, we observed that this trend of decline in the no. of imaging investigations was observed in the rest of the world as well.^8^-^10^ Similarly in the studies described by our local authors there was a declining shift in the patient numbers across different departments in the other parts of country as well.^11^-^13^ There was a significant reduction in the number of elective procedures with emphasis placed on patients requiring emergency services, as we also observed in our own department.^14^,^15^

### Limitations of the study

Firstly this is a single center study and might not reflect the reality of other geographical distributions in Pakistan. Secondly, our data collection is dependent upon the Radiology Information system by entering specific codes for each radiological imaging. These codes are primarily ordered by primary physicians of the patients which although are counter checked by Radiology personal, however there might be underestimation in the true numbers that we have described in our study especially pertaining to subdivision of CTs for specific indications.

Hence radiology department, along with pulmonary medicine, was the binding force of the rapidly changing workflow system adopted by the healthcare systems worldwide to deal with the devastating implication of this global pandemic.^4^,^9^,^10^

## CONCLUSION

COVID-19 pandemic led to a reduction in overall radiology work volume particularly in the initial months, contributed by lockdown and movement restrictions. However, this was compensated by an increase in the number of studies performed through emergency and for management/screening of COVID-19 infection. Therefore, it is of utmost importance that in preparedness of such pandemics, early disease detection, exposure limitation, maintenance of adequate diagnostic aides as well as appropriate staff numbers are focused upon in order to handle unprecedented challenges presented. Since radiological imaging will keep on providing pivotal role in pandemic surges, time sensitive diagnostic imaging pathways should always be in place to provide quality care to patients irrespective of national/global circumstances.

### Author’s Contribution:

**LM:** Conceived, designed, did literature review, performed statistical analysis & drafted the Manuscript.

**SG:** Participated in analysis and interpretation of data and critically reviewed the manuscript.

**RS:** Helped in collection of data.

**SB:** Helped in collection of data, and critically revised the manuscript.

All authors provided final approval for publication of the manuscript and are responsible for the integrity of the study.
